# Artificial Intelligence Advances in Transplant Pathology

**DOI:** 10.3390/bioengineering10091041

**Published:** 2023-09-04

**Authors:** Md Arafatur Rahman, Ibrahim Yilmaz, Sam T. Albadri, Fadi E. Salem, Bryan J. Dangott, C. Burcin Taner, Aziza Nassar, Zeynettin Akkus

**Affiliations:** 1 Department of Laboratory Medicine and Pathology, Mayo Clinic, Jacksonville, FL 32224, USA; 2 Department of Mathematics, Florida State University, Tallahassee, FL 32306, USA; 3 Computational Pathology and Artificial Intelligence, Mayo Clinic, Jacksonville, FL 32224, USA; 4 Department of Transplantation Surgery, Mayo Clinic, Jacksonville, FL 32224, USA

**Keywords:** transplant pathology, artificial intelligence, kidney transplant, heart transplant, liver transplant, lung transplant, digital pathology

## Abstract

Transplant pathology plays a critical role in ensuring that transplanted organs function properly and the immune systems of the recipients do not reject them. To improve outcomes for transplant recipients, accurate diagnosis and timely treatment are essential. Recent advances in artificial intelligence (AI)-empowered digital pathology could help monitor allograft rejection and weaning of immunosuppressive drugs. To explore the role of AI in transplant pathology, we conducted a systematic search of electronic databases from January 2010 to April 2023. The PRISMA checklist was used as a guide for screening article titles, abstracts, and full texts, and we selected articles that met our inclusion criteria. Through this search, we identified 68 articles from multiple databases. After careful screening, only 14 articles were included based on title and abstract. Our review focuses on the AI approaches applied to four transplant organs: heart, lungs, liver, and kidneys. Specifically, we found that several deep learning-based AI models have been developed to analyze digital pathology slides of biopsy specimens from transplant organs. The use of AI models could improve clinicians’ decision-making capabilities and reduce diagnostic variability. In conclusion, our review highlights the advancements and limitations of AI in transplant pathology. We believe that these AI technologies have the potential to significantly improve transplant outcomes and pave the way for future advancements in this field.

## 1. Introduction

According to the Centers for Disease Control and Prevention (CDC), the most commonly transplanted organs in the USA are the kidneys, liver, heart, lungs, pancreas, and intestines. Although there are approximately 100,000 people waiting for organ transplants every day, organ supplies remain limited. An estimated 14,000 deceased organ donors are available, each providing an average of 3.5 organs, while living donors supply only 6000 organs each year [1]. In the standard of care for organ transplantation, both donors and recipients undergo a pre-transplant assessment of histocompatibility, pathology, and clinical case histories. When a matched pair is found, the organ transplantation will proceed and then subsequently be monitored. Post-transplant monitoring includes electronic medical record (EMR) review, blood and body fluid assessment for organ function and formation of donor-specific antibodies, and protocol biopsies if there is a suspicion of rejection.

Recent advancements in deep learning-based AI approaches have revolutionized the field of digital pathology by enabling the development of AI-empowered diagnostic models for analyzing digitized glass slides of biopsy specimens. AI-empowered digital pathology can be extremely helpful in transplant pathology, as it could reduce inter-reader variability between pathologists, allow teleconsultation for pre and post transplantation, and provide a second opinion as well as assessing several morphological parameters and their spatial relationships. Over the years, several AI models have been developed for assessing transplant-related heart, lung, kidney, and liver pathologies [2,3,4,5]. Transplant pathology is a highly specialized field in which AI-empowered digital pathology tools could aid pathologists in making better decisions and eliminating/reducing the diagnostic variability among them. In addition to AI advances in transplant pathology, AI has also been used in organ allocation and donor-recipient matching [6,7,8,9], transplant oncology [10], and immunosuppressive therapy [11].

**Figure 1:**
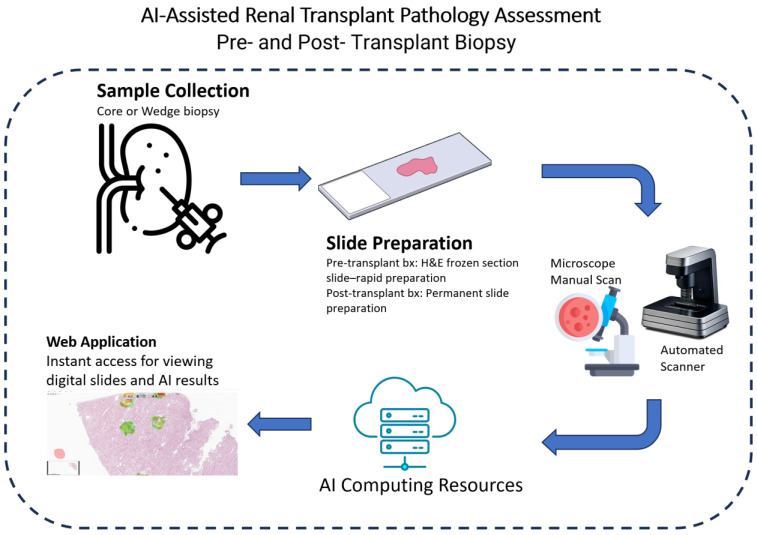
A depiction of an AI-assisted renal transplant pathology workflow.

In spite of the impressive success of AI models in integrating information from multi-modality data sources (e.g., histopathological reports, lab test results, radiological features, and patient demographics), our focus in this review is on the AI-empowered analysis of whole-slide images (WSIs), which is the cornerstone of diagnosis and prognosis in transplant pathology. Within the scope of this review, we aim to present the advancements of AI in the diagnosis and prognosis of transplant-related heart, lung, liver, and kidney pathologies. Furthermore, we offer insights into the future trajectory of AI-assisted approaches in the domain of transplant pathology. A flowchart of AI-assisted transplant pathology is depicted in Figure 1. An example of a web-based framework that could be used for transplant pathology is suggested in Akkus et al. [12] 

## 2. Methods 

### 2.1. Literature Search Strategy

We performed a thorough analysis of the literature using the Web Of Science and PubMed search engines. We included peer-reviewed journal publications and conference proceedings addressing the application of deep learning-based AI to transplant pathology before 30 April 2023. This systematic review was conducted using the Preferred Reporting Items for Systematic Reviews and Meta-Analysis (PRISMA) checklist [13]. The details of the search and our systematic review protocol are registered in the OSF public registries of systematic reviews: https://doi.org/10.17605/OSF.IO/XFGZN, (accessed on 1 May 2023).

### 2.2. Study Inclusion and Exclusion Criteria

**Figure 2:**
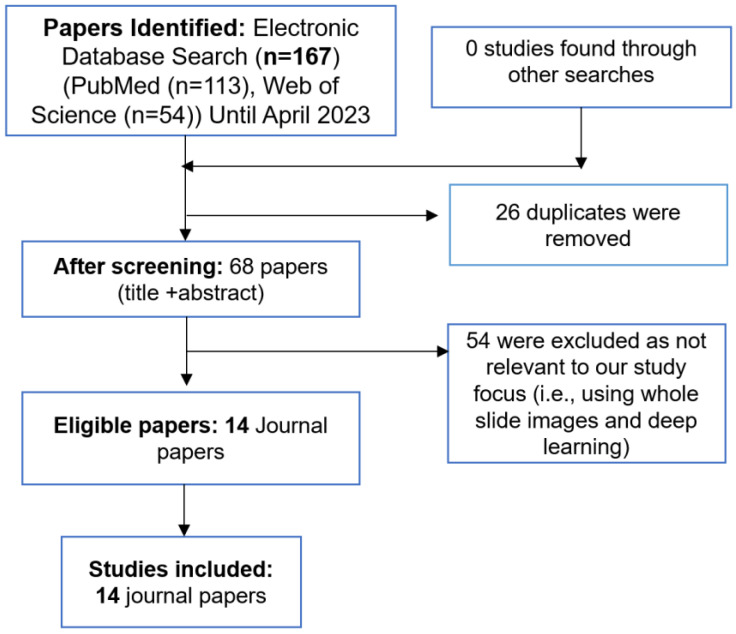
An overview of the systematic review process.

We included all full-text articles focusing on deep learning-based artificial intelligence in transplant pathology. We excluded studies using classical machine learning algorithms that rely on hand-crafted feature extraction. Furthermore, we also searched reference lists of retrieved articles and reviewed articles in the field to identify eligible studies that met inclusion and exclusion criteria. MAR and ZA reviewed titles and abstracts. To facilitate a structured analysis, the gathered reports were categorized into four distinct groups based on the transplanted organs: heart, lungs, kidneys, and liver. (See Figure 2 for a detailed flowchart used for identification, screening, eligibility, and inclusion).

## 3. Results

The initial systematic literature search yielded a total of 167 relevant articles. After removing duplicates, a rigorous selection process resulted in the inclusion of 14 journal papers that aligned with the defined scope and objectives of this review.

### 3.1. AI Advances in Transplant Heart Pathology

Cardiac allograft rejection is a critical concern in heart transplantation, primarily due to the limited availability of donor organs. Although endomyocardial biopsy with histopathology grading is the standard of care for diagnosing cardiac allograft rejection, substantial inter- and intra- observer variability among pathologists may result in inappropriate treatment with immunosuppressive drugs, redundant follow-up biopsies, and deteriorated transplant outcomes.

To date, there were four studies that discussed the use of artificial intelligence to assess cardiac allograft rejection and survival prediction. Giuste et al. [14] studied synthetic image generation to improve the risk assessment of rare pediatric heart transplant rejection. They trained progressive and inspirational Generative Adversarial Networks (GANs) to generate high resolution synthetic images with rejection signs that helped improve the performance of their allograft rejection classifier model. Despite their limited dataset consisting of only 12 non-rejection and 12 rejection slides, their approach significantly improved the performance of their allograft rejection classifier model, which achieved an area under receiver operating curve (AUC) of 98.84% for image tile-based rejection detection and 95.56% for biopsy rejection prediction at the WSI level. Peyster et al. [2] presented an automated WSI analysis pipeline based on handcrafted feature extraction and selection for histological grading of cardiac allograft rejection. Their study cohort included 2472 biopsy slides from three major US transplant centers. Their model performance was comparable to pathologists’ agreement (65.9% vs. 60.7%). Although this study did not meet our inclusion criteria, we refer readers to this paper for comparison to deep learning-based AI models. A limitation of this study is that the ground truth diagnosis label was obtained based on scoring only one section of tissue digitally while pathologists examine multiple slide sections under the microscope to reach the consensus of a final grade. Later, Lipkova et al. [15] published a study based on deep learning for the assessment of cardiac allograft rejection from the WSIs of endomyocardial biopsies. Their model demonstrated allograft rejection with a notable AUC of 0.962, which is a significant improvement compared to the study of Peyster et al. [2] that used hand-crafted features and a classical machine learning approach. Additionally, the AUC for differentiating between low and high-grade rejection was reported as 0.83. In their pipeline, they used a pre-trained CNN model to extract features from image patches and fine-tuned on three fully connected layers and a separate classifier was trained to estimate the rejection grade. The model was trained on 80% of 1690 internal WSI image datasets and validated on two external datasets: 1717 WSI slides of 585 patients from Turkey and 123 WSI slides of 123 patients from Switzerland. Lastly, Glass et al. [16] fine-tuned a pre-trained VGG model to predict myocyte damage in cardiac transplant acute cellular rejection (ACR). The authors annotated 19,617 regions including 10,855 regions of ACR, 5002 of healing injury, and 3760 of normal from 200 H&E slides and reported 94% for validation accuracy. A summary of these studies is provided in Table 1.

**Table 1:** A summary of previous AI studies investigating transplant heart pathology.

Author, Year	Objective	AI Model	Dataset	Performance
Giuste et al., 2023 [14]	Enhancing risk assessment of rare pediatric heart transplant rejection through the generation of synthetic images	Progressive and inspirational GAN [17,18]	12 non rejection and 12 rejection slides	95.56% AUROC for biopsy level rejection detection with 83.33% sensitivity and 66.67% specificity
Lipkova et al., 2022 [15]	Assessment of cardiac allograft rejection from endomyocardial biopsies	Pre-trained deep residual CNN [19]	Training: 1352 WSI slides; Validation: 1840 WSI slides	Allograft reject detection with an AUC of 0.962
Peyster et al., 2021 [2]	Histological grading of cardiac allograft rejection	Computer-Assisted Cardiac Histologic Evaluation (CACHE) grader pipeline	2472 endomyocardial biopsy slides	Differentiate low- and high-grade rejection with an AUC of 0.83
Glass et al., 2020 [16]	Determine myocyte damage in cardiac transplant acute cellular rejection	Pre-trained VGG16 [20]	19,617 annotations (10,855 regions of ACR; 5002 healing injury; 3760 normal)	Detection of myocyte damage (Grade 1R2) from non-myocyte damage (Grade 1R1A) with 94% validation accuracy

### 3.2. AI Advances in Transplant Lung Pathology

Despite advances in the immunosuppressive therapies and immunosuppressive drugs used, one-third of lung transplant recipients experience at least one episode of treated acute rejection in the first year after transplantation according to the report of the registry of the International Society of Heart and Lung Transplantation [21]. However, contrasting findings from the Organ Procurement and Transplantation Network/Scientific Registry of Transplant Recipients indicate a lower incidence of less than one-fifth in the first year [22]. Gholamzadeh et al. [23] published an in-depth systematic review about classical machine learning-based techniques to improve lung transplantation outcomes and complications. However, we exclusively focused on deep learning-based AI studies in our review and refer readers to their paper for further information. In one notable study, Davis et al. [3] investigated detecting acute cellular rejection (ACR) in lung transplant biopsies using AI. Board-certified lung transplant pathologists annotated a total of 3349 annotations (2580 regions of normal, 769 lesions of A1/A2 rejection). They included 614 A1/A2 lesions and 2064 regions of normal for the training set. On the other hand, the validation set included 155 A1/A2 lesions and 156 regions of normal to evaluate their AI model performance. Remarkably, their AI model distinguished the vascular component of ACR from normal alveolated lung tissue with 95% validation accuracy. Throughout our search, we identified only one study about lung transplant pathology using AI and one study about donor-recipient matching using AI [24]. Davis et al. presented promising results in identifying ACR, which is related to chronic lung allograft rejection, in lung transplant patients. The main limitation of their research is the lack of multi-institutional validation testing.

### 3.3. AI Advances in Transplant Kidney Pathology

Kidney transplantation is the most frequently performed solid organ transplantation worldwide. According to the United Network of Organ Sharing (UNOS), more than 25,000 people in the USA had kidney transplants performed in 2022, which was 3.4% more than in 2021 [25]. Despite the growing demand for kidney transplantation, the field of pathology is facing a decline in the available workforce. As per the Organ Procurement and Transplantation Network (OPTN), there are 88629 patients in the US currently on the waiting list for a kidney transplant as of July 23, 2023 [26]. Based on transplants conducted between 2008 and 2015, the 5 years post-transplant survival rates for males and females are 85.85% and 88.2%, respectively [26]. Although leveraging AI is new in this field, it could be useful in many ways such as finding a better matchmaking process between donors and patients, assessing histopathology of kidney biopsies, and guiding the treatment and management of transplant patients. The most common kidney biopsy scoring systems are Remuzzi [27], Banff [28], Leuven [29], and the Maryland Aggregate Pathology Index (MAPI) [30,31]. Table 2 provides a comparative overview of these models. The application of AI in transplant kidney pathology has been examined in a number of studies [4,32,33,34].

**Table 2:** Comparison of kidney biopsy scoring system models [30,35,36,37].

	Remuzzi	Banff	Leuven	MAPI
Scoring System	Scoring range between 0–12	3 grades (Mild, Moderate, and Severe) and 6 rejection categories	Score > 60 or Score < 60	Scoring range between 0–15
Selection Criteria	Glomerulosclerosis, tubular atrophy, interstitial fibrosis, and arterial narrowing	Vascular and other histologic abnormalities	Glomerulosclerosis, donor age, interstitial fibrosis, and tubular atrophy	Histologic parameters (glomerular sclerosis, arteriolar hyalinosis, cortical scar, and periglomerular fibrosis)

Hermsen et al. [32] employed a UNet [38] architectural CNN model for multiclass segmentation of digitized kidney biopsy tissue sections with periodic acid-Schiff (PAS) staining. The model was trained on 40 WSIs and validated 10 WSIs from their home institution and 10 WSIs from an external institution. In another study, Hermsen at al. [33] trained a UNet model to quantify inflammatory and chronic features in kidney transplant biopsies. Kers et al. [34] investigated the performance of multiple CNN architectures to predict transplant rejection. The study encompassed retrospective multicentered data, including 5844 digital whole-slide images of kidney allograft biopsies obtained from 1948 patients. A 3-fold cross-validation approach was employed, and an external dataset consisting of 101 WSIs was used for evaluation. The limitations of this study encompass its concentration on Western European institutions, lack of ethnicity data due to legal restrictions, absence of certain baseline characteristics, and restricted staining variability. Smith et al. [4] used a binary thresholding approach and trained a UNet model for glomeruli segmentation to assess interstitial inflammation from CD45-stained digital slides. They included a total of 60 biopsies from 53 patients in their study and observed a strong correlation between their automated inflammation scoring and Banff scoring. This study’s limitations include focusing on pixel counting rather than cell counting, potential complexity in implementing a deep learning approach, and a retrospective design with limited statistical power. Wilbur et al. [39] trained a modified version of the AlexNet CNN model to identify glomeruli on renal biopsies containing four stains (H&E, trichrome, silver, and PAS) from multiple institutions. The study encompassed 71 biopsies that were split into training/validation (*n* = 52) and testing (*n* = 19). The authors emphasized the importance of diverse datasets for developing generalizable AI models. The sensitivity of their model ranged from 90% to 93% for the intra-institutional dataset vs. 77% for the inter-institutional dataset. A summary of these studies is shown in Table 3. Furthermore, several review papers discussing AI in kidney transplantation and the assessment of renal transplant prognosis using classical machine learning approaches are available for interested readers in the additional resources [27,40,41,42,43,44,45].

**Table 3:** Summary of previous AI studies on transplant kidney pathology.

Author, Year	Objective	AI Model	Dataset	Performance
Hermsen et al., 2019 [32]	Multiclass segmentation of digitized kidney biopsy tissue sections	UNet [38]	Training: 40 WSIs; Validation: 20 WSIs (Home: 10; External Institution: 10)	Detected 92.7% of all glomeruli in nephrectomy samples with 10.4% false positives
Hermsen et al., 2022 [33]	Quantifying the chronic and inflammatory lesions in kidney transplant biopsies	UNet [38]	125 WSI pairs of periodic acid-schiff- and CD3-stained slides	The tissue class glomeruli was segmented with precision, recall, and dice scores of 0.96, 0.94, 0.95, respectively
Kers et al., 2022 [34]	Classifying histology of kidney allograft biopsies	Single CNN (InceptionV3) [46], Serial CNN	5844 WSIs from 1948 patients	AUROC (Single CNN) of 0.86, 0.78, and 0.70 for the normal and rejection disease classes, respectively
Smith et al., 2023 [4]	Quantifying the amount of non-glomerular inflammation within the cortex	UNet [38]	60 biopsies from 53 patients	Precision, recall, and dice scores for glomeruli identification were 0.888, 0.830, and 0.858, respectively
Wilbur et al., 2021 [39]	Identifying glomeruli on renal biopsy containing four stains from multiple institutions	Modified version of AlexNet	71 biopsies (Training: 52; Testing: 19)	Sensitivity of 90–93% for intra-institutional and 77% for inter-institutional dataset

### 3.4. AI Advances in Transplant Liver Pathology

Like other organ transplants, Liver Transplantation (LT) is a crucial treatment for patients with end-stage liver diseases. Recent advancements in surgical techniques, improved management of immunosuppressive drugs, and enhanced understanding of post-transplant morbidities have led to a significant increase in LT procedures. This surge in demand, however, has also led to a shortage of organ donors, resulting in a substantial waiting list for LT candidates [47]. Despite the disparity between organ supply and demand, over a third of donor livers are being rejected due to the risk of early allograft dysfunction (EAD), based on histopathologic findings [48]. The management of LT is complex, and the current approaches are not sufficient in clinical decision making. So, a data-driven LT could be useful in both pre- and post- LT settings [48,49]. Although this research area is quite new, several studies have already been conducted. Narayan et al. [50] published research regarding the use of AI for predicting donor liver allograft steatosis and early post-transplantation graft failure. They developed a Computer Vision AI platform (CVAI) to score donor liver steatosis and compared its capability for predicting EAD against pathologist steatosis scores. The study included liver biopsy slides data from 2014 to 2019 consisting of 25,494 images from 90 liver biopsies. The results indicated that the CVAI platform demonstrated slightly better calibration scores than pathologist steatosis scores. Their study was chiefly limited by a small sample of donor liver from a single institution and the presence of selection bias in the test for association with EAD. Yu et al. [51] designed a Multiple Up-sampling and Spatial Attention guided UNet model (MUSA-UNet) to segment liver portal tract regions in liver WSI that correlates with the stage of liver fibrosis. The dataset consisted of 53 WSIs, 30 of which were used for training and 23 for testing. They obtained an average of 0.94 precision, 0.85 recall, 0.89 F1 score, 0.89 (accuracy), and 0.80 (Jaccard Index) for their model MUSA-UNet. The major limitation of their study is the need for a more diverse training dataset with stain variations and annotations from multiple pathologists at various institutions.

Lu et al. [5] proposed an improved deep learning classifier (MobileNetV2_HCC_class) that could predict hepatocellular carcinoma (HCC) recurrence after liver transplantation. Their study was conducted on 1118 patients where 642 patients were used for training, 144 for testing, and 302 for validation. The hazard ratio obtained from the classifier in the LT set was 3.44 (95% CI 2.01–5.87, *p* < 0.001) and 2.55 (95% CI 1.64–3.99, *p* < 0.001) when known prognostic factors were adjusted.

Sun et al. [52] developed a deep learning model constructed from the pretrained VGG16 architecture [20] to estimate the percent steatosis in donor liver biopsy frozen sections. Their model generated a probability map from an input WSI to the output percent steatosis. Their research dataset comprised 96 WSIs, with 30 slides allocated for training and 66 for testing purposes. During the testing phase, their AI model demonstrated a notable correlation coefficient (r) of 0.85 and intraclass correlation coefficient (ICC) of 0.85, both of which surpassed the on-service pathologist’s performance (r = 0.74 and ICC = 0.72). A summary of these studies can be found in Table 4.

**Table 4:** Summary of previous AI studies on transplant liver pathology.

Author, Year	Objective	AI Model	Dataset	Performance
Narayan et al., 2022 [50]	Prediction of donor liver allograft steatosis and early post-transplantation graft failure	CVAI model consisting of Fully Convolutional Networks (FCN) [53] and UNet	25,494 images from 90 liver biopsies	CVAI peak mean IU 0.80; steatosis score median (CVAI 3% vs. pathologist 20%)
Yu et al., 2022 [51]	Segmentation of portal tract regions from whole-slide images of liver tissue biopsies	MUSA-UNet, FCN, UNet, DeepLab V3 [54]	53 WSIs (Training and Validation: 30; Testing: 20)	The precision, recall, and F1 score of MUSA-UNet were 0.94, 0.85, and 0.89, respectively
Liu et al., 2022 [5]	Predict HCC recurrence after liver transplantation	UNet, MobileNetV2_HCC_class	1118 patients (Training: 642; Testing: 144; Validation: 302)	Hazard ratio of 3.44 for LT set and 2.55 with adjusted known prognostic factors
Sun et al., 2020 [52]	Quantify percent steatosis in donor liver biopsy frozen sections	Pre-trained VGG16 (truncated at bottleneck layer)	96 WSIs (Training: 30; Testing: 66)	r = 0.85, ICC = 0.85 on testing samples

## 4. Discussion

In this comprehensive review, we have presented the latest advancements in AI-assisted transplant pathology, with a specific focus on the utilization of WSI. As we embark upon the emerging era of digital pathology coupled with the promising potential of AI technologies, therein lies a significant opportunity to revolutionize various aspects of solid organ transplantation. These advancements hold the capacity to positively impact organ procurement processes, optimize the dynamic adaptation of immunosuppressive drugs, and enhance graft and patient survival through effective post-transplant monitoring. Notably, numerous deep learning-based AI applications have emerged, catering to different transplant organs for the precise assessment of histopathology in tissue biopsies. The majority of these proposed AI models, however, have been trained primarily on relatively modest datasets and lack external validation. In order to ensure robust and reliable outcomes, it is imperative to have well-trained and rigorously validated AI models that assess the histopathology of transplant organ biopsies. With such AI models, the field will be able to gain valuable insights into the decision-making process surrounding rejection or acceptance of organs, possibly predict poor outcomes in advance, and reduce postoperative complications significantly. By enhancing patient care and outcomes in the area of solid organ transplantation, this development promises to elevate transplant pathology to new heights.

The histopathological evaluation of organ biopsies could play a crucial role in the decision-making process of accepting or discarding the organs of deceased donors for transplantation and in post-transplant monitoring if there is a suspicion of rejection. The major issue in the current practice of pre-transplant histopathology assessment is the low agreement between the pathologists [55]. The presence of wide variability and subjectivity among pathologists raises a big concern in biopsy scoring that leads to the suboptimal usage of organs. The issue of variability in pre-transplant biopsies can be attributed to the rapid embedding and fixation methods used for examination, resulting in frozen artifacts and generating lower quality slides. Conversely, permanent slides with more elaborate preparations are employed for post-transplant biopsy examinations. Despite the fact that histopathology assessment of transplant organ biopsy requires specialized training and expert consultancy, pre-transplant biopsies are frequently interpreted by on-call general pathologists on most occasions. We, therefore, believe that adaptation of digital pathology and exploiting AI and informatics tools could eliminate the wide variability and subjectivity among pathologists, provide a second virtual expert opinion, and improve clinical workflow efficiency. Embracing these advancements could shape a new era in solid organ transplantation, improving diagnostic accuracy, and helping patients in need of life-saving surgery.

The procurement of transplant organs primarily relies on donations from deceased or unrelated individuals. Therefore, immunosuppressive drugs are given to patients to increase tolerance to transplanted organs in the long term and prevent rejections. Monitoring significant histopathologic changes of allografts over time is essential in assessing transplant rejection and the refinement of immunosuppressive regimens to improve outcomes of transplant patients. In this context, the integration of AI-empowered digital pathology and EMR data proves valuable, as it can support informed decision making for tailoring individualized immunosuppression treatment regimens, thereby optimizing the management of transplant patients.

High-throughput digital pathology slide scanners have recently become available and are being adapted in clinical workflows. Concurrently, developments in computing hardware, cloud resources, AI tools, data storage, and network speed have facilitated more efficient processing of vast quantities of WSI data. Moreover, the availability of portable single slide scanners allows for rapid onsite evaluation of pathology slides which may prove beneficial in organ procurement scenarios. In the realm of pathology departments, digital pathology, automated image analysis of WSI with AI, and web/cloud-based applications have emerged which enables instant access and sharing of WSI and diagnostic reports. These technologies facilitate ready access to second opinions. The adoption of AI-assisted digital pathology workflows holds immense potential for improving patient outcomes and optimizing organ utilization in transplant pathology. Digital workflows, however, pose challenges due to their cost-intensive nature. Additionally, ensuring reproducibility and generalizability of AI models requires the development of datasets from multiple institutions and scanners. In addition, it is difficult to integrate existing AI tools effectively into clinical workflows at scale while maintaining efficiency. It will be essential to address these challenges and integrate AI-assisted digital pathology into transplant pathology to enhance patient care and organ preservation.

## 5. Conclusions

AI-empowered digital pathology is currently showing promise in facilitating the histopathological evaluation of organ biopsies, with the potential to mitigate the issues of variability and subjectivity encountered among pathologists. While AI applications in solid organ transplantation remain a nascent field, ongoing research is being conducted by investigators to explore its full potential. It is crucial to acknowledge that certain limitations, as previously discussed, warrant careful consideration and resolution before the widespread implementation of these AI tools in clinical workflows. Referring to the global aspect, cross-border organ transplantation presents significantly greater complexities due to a range of intricate challenges such as the illicit trafficking of human organs, variation of legal and regulatory frameworks, cultural and ethical differences, and financial and insurance matters, as well as travel and visa restrictions, etc. All of these factors need to be carefully addressed to ensure the safety, success, and ethical integrity of the procedures.
